# Evaluating Pathoma Use Among MSUCOM Students During Pre-Clinical Coursework and COMLEX-USA Level 1 Preparation

**DOI:** 10.51894/001c.164394

**Published:** 2026-08-01

**Authors:** Matthew Leung, Leonardo Tavarone, Nicholas Barron, Kirsten Waarala

**Affiliations:** 1 College of Osteopathic Medicine, Michigan State University

## 44

### Introduction

Osteopathic medical students frequently rely on third-party resources to supplement pre-clinical learning and prepare for COMLEX-USA Level 1. Pathoma is a widely used pathology resource, yet limited data describe how students integrate it longitudinally across coursework and licensing exam preparation.

### Objective

To characterize MSUCOM students’ longitudinal perceptions and patterns of Pathoma use during pre-clinical coursework and COMLEX-USA Level 1 exam preparation.

### Methods

Second-year MSUCOM students volunteered to participate. Forty students were selected via random number generation from a pool of 98 volunteers, with representation across MSUCOM sites. Participants completed Qualtrics surveys at the end of semesters 3 through 5 assessing Pathoma use (format, frequency, time spent, targeted topics/courses, and barriers). After Level 1, participants completed a semi-structured Zoom interview with an investigator (LT) exploring Pathoma’s perceived strengths and limitations, overall perceived value, comparisons with other resources, and peer influences.

### Results

During pre-clinical coursework, 18/30 (60%) interviewees reported rarely or never using Pathoma, whereas 28/30 (93%) reported using Pathoma during Level 1 exam preparation. In the semester 3 survey, 18/38 (48%) respondents preferred the online format whereas post COMLEX-USA Level 1, 21/30 (70%) preferred online use. Average reported coursework use was 7 hours. Of the interviewees using Pathoma, 23/28 (82%) increased average use. Students most commonly emphasized early chapters 1–3 and frequently used Pathoma alongside other resources, particularly First Aid (14/30, 47%), UWorld (9/30, 30%), and Sketchy (15/30, 50%). A commonly cited limitation was the absence of an integrated question bank. 16/30 (53%) interviewees reported increased confidence for Level 1 attributable to Pathoma, although a minority described the content as dense or less engaging. Most interviewees indicated they would recommend Pathoma to junior students.

### Discussion

In this longitudinal cohort, Pathoma was used infrequently during routine coursework but was commonly adopted during COMLEX-USA Level 1 preparation. Students perceived Pathoma as a quality resource for conceptual reinforcement and high-yield pathology review, especially when paired with question-based resources. These findings may inform how institutions counsel students on resource selection and better align curricular supports with learners’ study behaviors.

